# Towards the invasion of wild and rural forested areas in Gabon (Central Africa) by the Asian tiger mosquito *Aedes albopictus*: Potential risks from the one health perspective

**DOI:** 10.1371/journal.pntd.0011501

**Published:** 2023-08-16

**Authors:** Judicaël Obame-Nkoghe, David Roiz, Marc-Flaubert Ngangue, Carlo Costantini, Nil Rahola, Davy Jiolle, David Lehmann, Loïc Makaga, Diego Ayala, Pierre Kengne, Christophe Paupy

**Affiliations:** 1 Laboratoire de Biologie Moléculaire et Cellulaire, Département de Biologie, Université des Sciences et Techniques de Masuku, Franceville, Gabon; 2 Unité de Recherche en Écologie de la Santé, Centre Interdisciplinaire de Recherches Médicales de Franceville, Franceville, Gabon; 3 MIVEGEC, Univ. Montpellier, CNRS, IRD, Montpellier, France; 4 Agence Nationale des Parcs Nationaux, Quartier Haut de Gué Gué, Libreville, Gabon; 5 Biological and Environmental Sciences, University of Stirling, Stirling, United Kingdom; Colorado State University, UNITED STATES

## Abstract

**Background:**

Since its first record in urban areas of Central-Africa in the 2000s, the invasive mosquito, *Aedes albopictus*, has spread throughout the region, including in remote villages in forested areas, causing outbreaks of *Aedes*-borne diseases, such as dengue and chikungunya. Such invasion might enhance *Ae*. *albopictus* interactions with wild animals in forest ecosystems and favor the spillover of zoonotic arboviruses to humans.

The aim of this study was to monitor *Ae*. *albopictus* spread in the wildlife reserve of La Lopé National Park (Gabon), and evaluate the magnitude of the rainforest ecosystem colonization.

**Methodology:**

From 2014 to 2018, we used ovitraps, larval surveys, BG-Sentinel traps, and human landing catches along an anthropization gradient from La Lopé village to the natural forest in the Park.

**Conclusions:**

We detected *Ae*. *albopictus* in gallery forest up to 15 km away from La Lopé village. However, *Ae*. *albopictus* was significantly more abundant at anthropogenic sites than in less anthropized areas. The number of eggs laid by *Ae*. *albopictus* decreased progressively with the distance from the forest fringe up to 200m inside the forest. Our results suggested that in forest ecosystems, high *Ae*. *albopictus* density is mainly observed at interfaces between anthropized and natural forested environments. Additionally, our data suggested that *Ae*. *albopictus* may act as a bridge vector of zoonotic pathogens between wild and anthropogenic compartments.

## Author summary

Since its first record in urban areas of Central Africa in the 2000s, the anthropophilic and invasive mosquito, *Aedes albopictus*, has continued to spread across remote rural areas and has promoted outbreaks of *Aedes*-borne diseases, such as dengue, chikungunya and Zika. Such invasion might enhance *Ae*. *albopictus* interactions with wild animals in forests neighboring villages and favor the spillover of zoonotic arboviruses to humans.

In this study, we reported the sustained spread of the *Ae*. *albopictus* in the wildlife reserve of La Lopé National Park (LNP, Gabon), and we evaluated the magnitude of its colonization of this rainforest ecosystem. Using various sampling methods between 2014 and 2018, we detected *Ae*. *albopictus* presence in wild forest galleries up to 15 km away from La Lopé, the main inhabited village within the park limits. We demonstrated that *Ae*. *albopictus* can invade forest ecosystems where the human presence is low or absent. These data indicate that *Ae*. *albopictus* may interact with wild animals and act as a bridge vector of zoonotic pathogens between the wild and anthropogenic compartments. However, our results showed that *Ae*. *albopictus* population density is higher at interfaces between the village and the forest. This suggests that this species might be present at the interface between anthropized and wild environments where the probability of contact with wild animals is increased, and consequently the likelihood of contributing to sylvatic and bridge transmission of pathogens. Our results also suggested that the presence of humans facilitates the colonization of natural forested habitat by this invasive species.

## Introduction

In addition to *Aedes aegypti*, the Asian tiger mosquito *Aedes* (*Stegomyia*) *albopictus* (Skuse, 1894) [1] is becoming a significant arbovirus vector, and has been implicated in the transmission of dengue (DENV), chikungunya (CHIKV) and Zika (ZIKV) viruses. These arboviruses cause diseases of important public health concern in the world, particularly in Central Africa where *Ae*. *albopictus* currently plays the primary vector role [2]. *Aedes albopictus* is an invasive vector species native from Asia that has spread worldwide [3,4]. It has been estimated that 6.3 billion people currently live in areas that are suitable for this species, thus increasing the potential risk of arboviral transmission [5]. Moreover, *Ae*. *albopictus* progressive expansion around the world (facilitated by global trade) highlights its success as an invasive species, thanks to its capacity to develop desiccation-resistance eggs, to use human-made containers for egg laying, to develop diapause in temperate areas [6], and its capacity to adapt to urban, peri-urban and forested environments where it can persist durably [7].

In Central Africa, *Ae*. *albopictus* is well established in several urban areas, and its expansion to remote areas (e.g., rural villages) is increasingly reported [8]. It might also durably invade forest ecosystems, as suggested by its repeated detection in some forested habitats in continental Africa [9]. In Central Africa, *Ae*. *albopictus* was first reported in the early 2000s in Cameroon [10], and has gradually spread across almost the entire region [11–13]. Now, *Ae*. *albopictus* is suspected to be the major *Stegomyia* species in several urban and rural areas in Central Africa, and a first-line vector for arboviruses [14]. For instance, after its introduction in Gabon in the 2000s [15], it rapidly spread across the country, favoring major CHIKV, DENV and ZIKV outbreaks in urban [16,17] and in remote rural settings [8]. Moreover, *Ae*. *albopictus* has the ability to invade new areas, exhibit vector competence for many viruses [18], and display broad feeding habits by consuming blood from various animals. It can also disrupt the epidemiological equilibrium of arbovirus transmission by selecting strains with enhanced virulence [19]. As a result, *Ae*. *albopictus* might establish in forest ecosystems or at their edges and interact with sylvatic cycles of pathogens. More worryingly, particularly for human health, *Ae*. *albopictus* presence in forests or at the forest margins could favor contact with potential reservoirs of enzootic arboviruses and thereby promote viral spillover to humans [18].

During its recent intercontinental range expansion, *Ae*. *albopictus* has mainly invaded anthropogenic habitats where it can exploit man-made containers and humans as blood source to become an efficient vector for epidemic arboviruses, such as DENV and CHIKV [20–22]. However, it is not clear whether the species has conserved its ancestral capacity to colonize sylvatic environments, particularly in newly colonized areas in tropical America and Africa [2,7]. In Asia, it is currently thought that *Ae*. *albopictus* is more adapted to forest margins (ecotones), the transition from forest to degraded or secondary forests, and open grasslands or scrub rather than to deep primary forests [7]. Recent studies have confirmed that *Ae*. *albopictus* has retained its ancestral capacity to colonize forest environments by laying eggs in natural breeding sites and by feeding on non-domestic vertebrates [18]. In Brazil, *Ae*. *albopictus* was observed up to 500m from the forest margins and may be established in a degraded forest of the Manaus region [23]. Despite its detection in forests bordering in human villages [8,9], the penetration (i.e., the ability to enter natural forested environments for its biological needs, including host seeking, oviposition activity, and resting) and the invasion dynamics (i.e., the long-lasting establishment and spread in a new environment) of *Ae*. *albopictus* in forest ecosystems and more importantly, its persistence as sylvatic populations (i.e., in the absence of humans) have never been studied in Africa.

In Central Africa, natural parks are among the potential invasion territories for *Ae*. *albopictus*. These parks attract many visitors and are potential hotspots of arbovirus transmission from wildlife reservoirs to humans by zoo-anthropophilic mosquitoes, such as *Ae*. *albopictus*. In Gabon, La Lopé National Park (LNP) includes large forest-savanna mosaic areas and primary forest blocks, and might be a hotspot of arbovirus circulation (Table 1). Therefore, it is a suitable field of investigation to test whether *Ae*. *albopictus* can enter and settle permanently in forest ecosystems, through interactions with the wild fauna, and whether it represents a risk of zoonotic arbovirus transfer to humans. To test whether *Ae*. *albopictus* can enter and settle permanently in forest ecosystems we carried out a field investigation in La Lopé village and in the adjoining northern part of the LNP. The aim of this study was to evaluate the *Ae*. *albopictus* distribution and the degree of penetration into the forest ecosystem, from interfaces between inhabited areas and forest, and between forest and forest-savanna towards the deeper parts of the forest compartment.

**Table 1:** Arboviruses isolated from sylvatic *Stegomyia* species in Central Africa and possibly circulating in the LNP.

Genus	Virus	Wild animal groups in which the virus circulation was documented (isolation or serological evidences)	Vector or suspected *Aedes* vector species belonging to the *Stegomyia* subgenus
*Alphavirus*	Chikungunya	African monkeys, bats	*Aedes* from *africanus* group, *Aedes opok*
	Middelburg	Rodents	*Aedes* from *africanus* group, *Ae*. *opok*
	Semliki Forest	Birds	*Aedes aegypti*, *Aedes* from *africanus* group, *Ae*. *opok*
	Banbanki	Rodents	*Aedes* from *africanus* group, *Aedes from simpsoni group*
*Flavivirus*	Bagaza	Unknown	*Aedes* from *africanus* group
	Wesselsbron	Unknown	*Aedes* from *africanus* group, *Aedes metallicus*, *Ae*. *opok*
	Bouboui	African monkeys, rodents, antelopes	*Aedes* from *africanus* group, *Ae*. *opok*
	Yellow Fever	African monkeys	*Aedes* from *africanus* group, *Ae*. *opok*, *Ae*. *metallicus*
	Zika	African monkeys	*Ae*. *albopictus*, *Aedes africanus*, *Ae*. *opok*
	Yaoundé	Rodents	*Ae*. *aegypti*
	West Nile	Birds	*Aedes* from *africanus* group
	Saboya	Rodents	*Aedes* from *africanus* group
*Bunyavirus*	Bunyamwera	Unknown	*Aedes* from *africanus* group, *Ae*. *opok*
	Bozo	Unknown	*Aedes* from *africanus* group, *Ae*. *opok*
	Ar B 28215	Unknown	*Aedes* from *africanus* group
*Orbivirus*	Orungo	Unknown	*Aedes* from *africanus* group, *Ae*. *opok*
*Rhabdovirus*	Kamese	Unknown	*Aedes* from *africanus* group
Other	Mengo	Rodents	*Aedes africanus*, *Ae*. *opok*

Source: Adam & Digoutte, 2005. Pasteur Institute and IRD CRORA (http://www.pasteur.fr/recherche/banques/CRORAdatabase, Last access 2015-04-15) [24]. In gray: viruses isolated in Central Africa: Cameroon, Central African Republic, Republic of the Congo. Bouboui, yellow fever, and Zika viruses were recovered from African monkey species or closely related species (same genus) present in the LNP (genus *Cercopithecus*, *Colobus*, *Erythrocebus* and *Galago*)

## Methods

### Ethics statement

This study was authorized by the National Center of Scientific and Technical Research (CENAREST, Gabon) under the authorization N° AR0010/19/MESRS/CENAREST/CG/CST/CSAR and AR0020/14/CENAREST/CG/CST/CSAR.

### Study area

**Fig 1:**
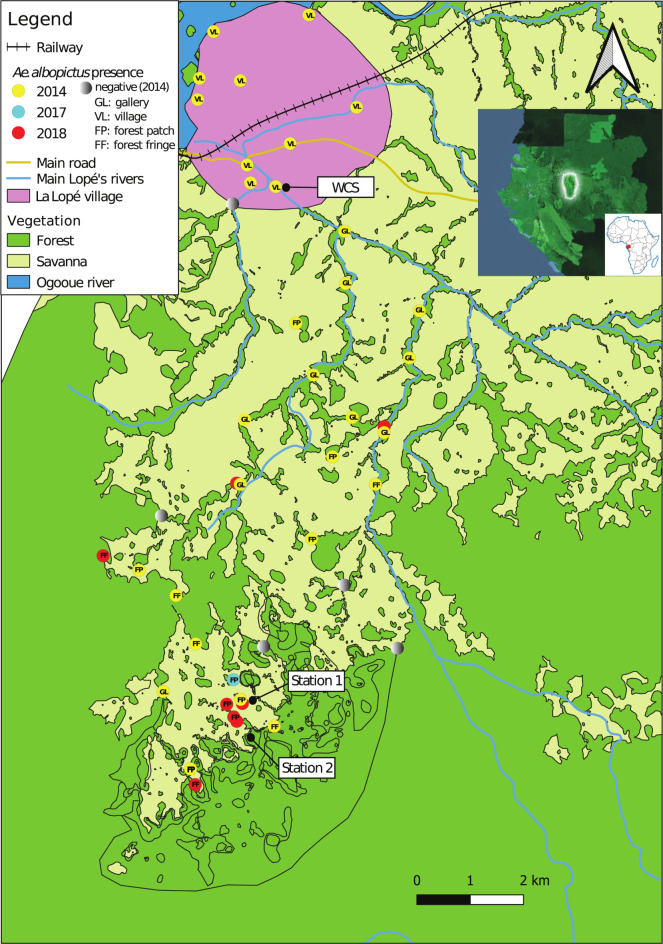
Study area and sites with *Ae*. *albopictus* occurrences between 2014 and 2018 in the LNP. “Negative (2014)” refers to ovitraps that were negative for *Ae*. *albopictus* in 2014. Black circles indicate the *WCS (site with neighboring habitations)*, *Station 1 (forest base camp)* and *Station 2 (forest with no human presence)* sites where ovitrap-based experiments were carried out along anthropization gradients to assess *Ae*. *albopictus* presence inside forest ecosystems. The base map was produced by digitizing the Gabonese land use map freely available from the *Agence Nationale d’Étude et d’Observation Spatiale du Gabon* (http://ageos.ga).

The LNP (central coordinates: 11.531397E, 0.517324S) covers 4,964km^2^ [25] of equatorial forest and is among the most important biodiversity hotspots in Gabon, with a very abundant and diversified wildlife [26]. The LNP is in an equatorial climate zone with temperatures ranging from 21°C to 28°C throughout the year. Rainfall ranges between 1,400mm and 3,800mm [27]. The northern part of the LNP is dominated by a forest-savanna mosaic landscape crossed by the Ogooué river and several smaller waterways along which La Lopé village is located (Fig 1). In this village with <2,000 inhabitants [27], the economic activity is essentially based on wildlife conservation and ecotourism. The majority of visitors from Gabon and other countries (~1,000 visitors per year) come by road or by train [28]. Further south, the forest-savanna mosaic landscape gives way to a dense equatorial forest with several forest-savanna ecotones.

### Study design and sampling procedure

**Fig 2:**
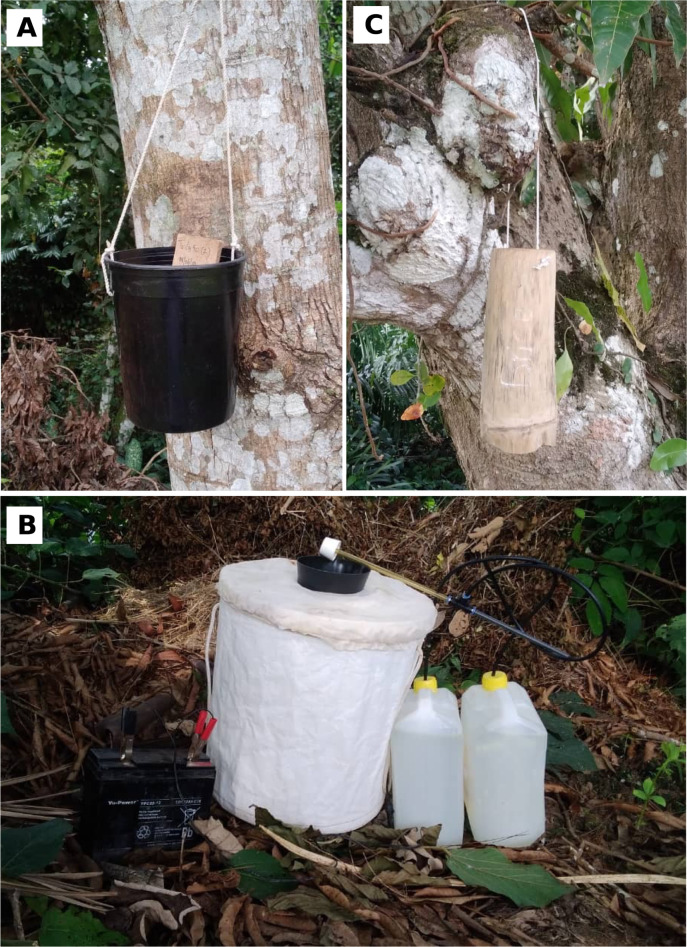
Sampling methods, including plastic ovitrap **(A)**, BG-sentinel trap **(B)**, and bamboo ovitrap **(C)**.

The different sampling periods corresponded to the long rainy season. Indeed, the LNP has a bimodal rainy season (long rainy and short rainy periods, from February to May and from October to November, respectively) interspersed with dry periods. First, to explore the range of *Ae*. *albopictus* dispersal from La Lopé village towards the wild compartment of the LNP, we carried out a sampling session using ovitraps and BG-Sentinel traps in May 2014. For ovitrap-based sampling, 35 spatial spots were randomly selected, in function of their accessibility, in the village (n = 10) and in the wild compartment southwards (n = 25), across the gallery forests and groves that are located within the wilderness area of the park and that separate the village and the station [this is the park’s observatory for biodiversity monitoring] (Fig 1). Collection sites were at least 300m apart and each contained five ovitraps left active for 14 consecutive days. Traps consisted of sections of bamboo trees (15-20cm long and 5-7cm in diameter) that contained 300mL of tap water and a 15cm x 5cm wood paddle for egg laying (Fig 2A). We hung these traps on tree trunks at 1m-1.5m from the ground. In the wild compartment of the park, we placed traps along the gallery forest network that connects the village to the forest block, and also in some selected isolated forest patches and at forest fringes (Fig 1). We deployed individual BG-Sentinel traps equipped with BG-lure and a carbon dioxide source from 8 am to 6 pm in the village (i.e., anthropic compartment; 1,340 hours of sampling effort) and in the wild side of the LNP (i.e., sylvatic compartment; 1,645 hours of sampling effort). For the carbon dioxide production, we used a home-made technique based on the adaptation of the Biogent system (https://eu.biogents.com/bg-co2-generator/). We used two mixtures, each containing 11 g of yeast, 1 kg of normal sugar [edible sugar], and tap water that were combined to obtain a final volume of 2.5 L in a 5 L plastic container. To connect the containers to the top center of the BG traps, we employed 5mm diameter tubes (Fig 2B). To follow the dynamics of *Ae*. *albopictus* invasion inside the park, we completed the dataset with additional occurrences reported for the species from 2017 and 2018 during *Ae*. *aegypti* surveys [29–31]. These observations were obtained using several sampling methods, including larval prospections in natural water collection sites (e.g., rock pools, tree holes), domestic (voluntarily filled by humans, e.g., drinking water, storage) or peri-domestic (abandoned man-made containers that are filled by rainfall) water containers, bamboo or plastic ovitraps (Fig 2C), BG-Sentinel traps, and human landing catches (HLC) (Fig 2D). For HLC, volunteers were posted at different capture sites. Upon landing on the volunteer’s bare legs, mosquitoes were captured using dry hemolysis tubes that were sealed with cotton. Then, mosquitoes were euthanized at -20°C for identification. Only volunteers already vaccinated against yellow fever were admitted to perform HLC. Additionally, consent was signed by each volunteer before the sampling. Although yellow fever vaccine is the only prevention against arbovirus risk, each volunteer also received anti-malaria prophylaxis to minimize the risk of malaria exposure.

Second, to assess *Ae*. *albopictus* colonization level in the forest ecosystem (i.e., its “capacity to exploit forest environments for suitable oviposition sites” [23]), in April 2019, we carried out a field experiment in the village and wild compartment of the park. Three sampling sites were selected to assess whether human activity or presence influences the colonization of forest ecosystems by *Ae*. *albopictus*. The sites were selected and spatially distributed based on a relative anthropization gradient: "inhabited forest edge" (*WCS*: in the village, a section of 30 neighboring habitations), "sparsely inhabited forest edge" (*Station 1*: forest base camp, less than 5 human dwellings housing 2–10 people who permanently live or work at this site), and "non inhabited forest edge" (*Station 2*: no human habitation) (Fig 1). In this study, “anthropization” refers to human presence or frequentation, as evidenced by human dwellings or traces of human passage or activities. We considered that a site was anthropized (or inhabited) when there was at least one human habitation within. 300m from the collection site. At the interfaces between anthropic and forest compartments (*WCS* and *Station 1*) and between savanna and forest (*Station 2*), we deployed ovitraps from the fringe towards the deeper parts of the forest compartment. We surveyed the colonization by *Ae*. *albopictus* by monitoring along the transects the number of eggs laid by females in ovitraps placed at different distances from the edge towards the forest. At each site, we set ovitraps at the intersections of a grid of parallel lines spaced 25m apart and crossed perpendicularly by parallel lines spaced 25m apart. At each site, the grid extended from the edge (0m) to 150m (*WCS*), 125m (*Station 1*), and 175m (*Station 2*) inside the forest compartment, depending on the spatial conformation of the forest blocks (S1 Fig). Ovitraps consisted of dark plastic cups that contained 300mL of mango leaf infusion and a 15cm x 5cm hardboard paddle for egg collection. In total, we deployed 105 ovitraps (35 at *WCS*, 30 at *Station 1*, and 40 at *Station 2*). At each site, we monitored oviposition for 10 consecutive days with a change of paddles after 5 days. We transported the recovered paddles to the insectary for 3 days of drying and egg counting. Then, we immersed wood paddles separately in 1 L distilled water in a plastic tray (40cm x 30cm x 7cm) for egg hatching. After 3 days of immersion, we removed the paddles from the trays and air-dried them at room temperature for 3 additional days before immersion in new distilled water following the same procedure. We fed mosquito larvae 0.01 g *TetraMin* fish food (Spectrum Brands, Inc.) every day and reared them to the adult stage. We morphologically identified adult mosquitoes using “custom” taxonomic keys based on updates of the Edwards’ identification keys for Ethiopian mosquitoes [32] and the Huang’s key for the subgenus *Stegomyia* of *Aedes* mosquitoes from the Afrotropical Region [33].

### Statistical analysis

All statistical analyses were carried out using the R software, v3.6.1 (https://www.r-project.org/). Assessment of *Ae*. *albopictus* dispersion from the village towards the wild compartment of the park was based on its presence at collection sites. To assess the level of colonization of the forest ecosystem, we used count data from the transect experiment. We used the Chi-squared test to compare the percentages of mosquitoes that developed from the egg to the adult stage. As the number of emerged adult mosquitoes was not equal to the number of collected eggs in the field due to egg viability, we used the weighted number of *Ae*. *albopictus* eggs (WNAE) as the main variable of interest. The WNAE is an estimation of the number of *Ae*. *albopictus* eggs collected relative to the percentage of *Ae*. *albopictus* adults after emergence. We calculated the WNAE as the frequency of *Ae*. *albopictus* adults multiplied by the total number of eggs collected in the field for each collection site. Then, we tested various generalized linear models to fit the penetration into the forest by *Ae*. *albopictus* using the "WNAE" as the response variable, and the "Distance" of the trap to the forest edge and the "Habitat" as fixed and random effects, respectively. We did not include data from *Station 2* in the models because of insufficient number of positive traps. We checked the WNAE distribution normality visually and using the Shapiro-Wilk normality test. We tested different models with a log link function and two alternative error structures: Poisson or negative binomial, using the *stats*, *glmmADMB*, *lme4*, and *MASS* packages [34–36]. Then, we used the *predict* function for model visualization and interpretation. We used the *Akima* package to interpolate in two dimensions the spatial distribution of the density of *Ae*. *albopictus* eggs in ovitraps [37]. We used the Kruskal-Wallis test to compare the mean number of *Ae*. *albopictus* eggs laid in each ovitrap among sites.

## Results

### Persistent invasion of uninhabited environments in La Lopé

We collected *Ae*. *albopictus* eggs at all ten collection points monitored in the village in 2014 (100%), confirming that the species was well established and widely distributed across the village (i.e., human compartment) (Fig 1). Moreover, adult collections with BG-Sentinel traps in the village (1,340 hours of sampling effort) showed that *Ae*. *albopictus* was the dominant species compared with other native *Aedes* species from the *Stegomyia* subgenus (Table 2). In addition, the HLC results of human landing collections confirmed that *Ae*. *albopictus* was the most abundant diurnal mosquito feeding off humans. In the forest compartment, 20 of the 25 ovitrap collection sites surveyed in 2014 (80%) were positive for *Ae*. *albopictus* (Table 2). This clearly showed that *Ae*. *albopictus* had widely spread throughout the northern part of the LNP, including sites with few or no human presence. The 20 positive sites were along interconnected gallery forests (n = 10), in forest patches (n = 4), and at deep forest fringes (n = 6) over large expanses in the national park, ranging from 1 to 15km away from the village (Fig 1). Occasional observations in 2017 and 2018 confirmed the persistence of *Ae*. *albopictus* over several years in the sylvatic compartment (Fig 1).

**Table 2:** Abundance and proportion of *Aedes* mosquito species from the *Stegomyia* subgenus collected in the LNP in 2014.

	Anthropic compartment			Forest compartment			Both compartments
	**BG-Sentinel (%)**	**HLC (%)**	**Sub-total (%)**	**BG-Sentinel (%)**	**HLC (%)**	**Sub-total (%)**	
**Invasive species**							
*Ae*. *albopictus*	482 (56.1)	126 (87.5)	608 (60.6)	110 (39.1)	126 (88.1)	236 (55.7)	844 (59.1)
**Native species**							
*Ae*. *aegypti*	80 (9.3)	18 (12.5)	98 (9.8)	20 (7.1)	15 (10.5)	35 (8.3)	133 (9.3)
*Ae*. *africanus* group	10 (1.2)	0	10 [1]	13 (4.6)	0	13 (3.1)	23 (1.6)
*Ae*. *apicoargenteus* group	4 (0.5)	0	4 (0.4)	7 (2.5)	0	7 (1.6)	11 (0.8)
*Ae*. *dendrophilus* group	10 (1.2)	0	10 [1]	21 (7.5)	0	21 (4.9)	31 (2.2)
*Ae*. *fraseri*	2 (0.2)	0	2 (0.2)	11 [4]	2 (1.4)	13 (3.1)	15 (1.0)
*Ae*. (*Stegomyia*) spp.	271 (31.5)	0	271 [27]	99 (35.2)	0	99 (23.3)	370 (26.0)
**Total**	**859 (100)**	**144 (100)**	**1003 (100)**	**281 (100)**	**143 (100)**	**424 (100)**	**1427 (100)**

HLC: Human Landing Catch; Sampling efforts: BG-Sentinel (1,340 hours and 1,645 hours in anthropic and sylvatic compartments, respectively) and HLC (6 hours and 12 hours in anthropic and sylvatic compartments, respectively)

In the forest compartment, *Ae*. *albopictus* represented 37.2% of all collected *Stegomyia* species using BG-Sentinel traps (110 *Ae*. *albopictus* among the 296 *Stegomyia* specimens collected during 1,340 hours of sampling; Table 2), and 86.9% of all human-biting mosquitoes from this subgenus (252 *Ae*. *albopictus* among the 290 *Stegomyia* specimens collected; Table 2). This indicated that, *Ae*. *albopictus* conserved its human feeding habits, even after invading the forest environment where the human presence is limited or absent. It also confirmed *Ae*. *albopictus* presence in forest galleries and at the forest fringes in the northern part of the LNP, raising the question of its capacity to colonize areas deeper in the LNP primary forest ecosystems.

### Relative abundance and distance from the forest ecotone

To determine *Ae*. *albopictus* colonization level in the forest ecosystem, in 2019, we collected, using plastic ovitraps, mosquito eggs at three ecotone sites with contrasting characteristics (i.e., distance from the village, level of anthropization) along forest penetration transects (Fig 1 and Table 3). In total, we collected 4,940 eggs over all sites, 2,467 were collected at *WCS* (49.9%), 1,831 at *Station 1* (37.0%), and 642 at *Station 2* (13.1%). After egg hatching and larval development, 2,415 adult mosquitoes emerged and were morphologically identified. The global emergence success from egg to adult stage was 48.9%, but varied significantly in function of the collection site (Chi-squared = 165.1, df = 2, *p* < 0.001): 58% for *WCS*, 49% for *Station 1*, and 14% for *Station 2*. Among the 2,415 emerged adults, 1,431 (59.2%) came from *WCS*, whereas 893 (36.9%) from *Station 1* and 91 (3.9%) came from *Station 2*. A generalized mixed effects model in which the percentage of adult emergence (all species included) was the response variable showed that there was no effect of “distance from the forest edge” (fixed effect) (*p* = 0.7), regardless of the sampling site (random effect). This suggests that the percentages of adults at the three sites were not skewed, regardless of the sampling site or the penetration depth into the forest (which could reflect a realistic diversity and abundance of mosquitoes that exploited ovitraps deployed at the three sites).

**Table 3:** Eggs collected and adult emergence percentages at the three egg sampling sites.

Site	Total egg number	Number of adults and percentage (%)			WNAE*
		*Ae*. *albopictus*	Other species	Total	
**WCS**	2,467	1,187 (82.9)	244 (17.1)	1,431 (100)	2,045
**Station 1**	1,831	525 (58.8)	368 (41.2)	893 (100)	1,075
**Station 2**	642	9 (9.9)	82 (90.1)	91 (100)	63

**WNAE**: Total Weighted Number of *Ae*. *albopictus* eggs

**Fig 3:**
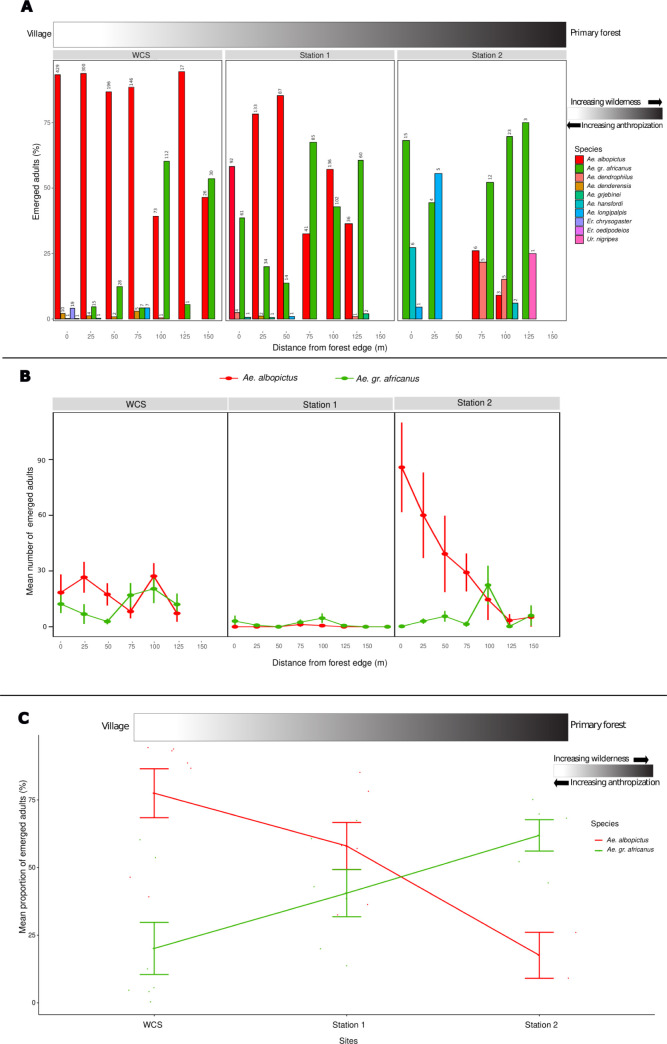
Relative abundance of *Ae*. *albopictus* and other species recovered from ovitraps. Mosquitoes are distributed along a gradient of anthropization from village to primary forest in La Lopé National Park, Gabon. **A:** Relative abundance of all species; the number of emerged adults at each distance sampled was added above the bars for each distance category. **B**: Mean number of emerged adults at each site. Species were limited to *Ae*. *albopictus* and *Ae*. *gr*. *africanus* because they were by far the most represented. **C:** Mean percentage of emerged adults for the two predominant taxa: *Ae*. *albopictus* and *Ae*. *gr*. *africanus* in three sites arranged according to their degree of anthropization.

Regarding the species relative abundance (determined as species percentage) according to the distance from the forest edge and sampling site (representing three levels of anthropization), we observed that, *Ae*. *albopictus* was the dominant species at *WCS* (high anthropization) at all distances except at 100m and 150m from the forest edge. It was followed by *Aedes* from the *africanus* group (*Ae*. gr. *africanus*) for which the relative abundance increased with the distance from the forest edge, and the highest relative abundances were at 100m and 150m from the forest edge (Fig 3A). At *Station 1* (low anthropization), *Ae*. *albopictus* was dominant from 0m to 50m and at 100m from the forest edge, followed by *Ae*. *gr*. *africanus* that displayed the highest relative abundances at 75m and 125m from the forest edge (Fig 3A). Conversely, at *Station 2* (no anthropization), *Ae*. *gr*. *africanus* was the most abundant, regardless of the distance from the edge, but not at 25m (Fig 3A). At all three sites, *Ae*. *albopictus* and *Ae*. *gr*. *africanus* were the two predominant species. Regarding the mean number of emerged adults, both species showed opposite trends regardless of the site, except at *Station 1* where both species were found in very few numbers at almost all distances from the forest edge. Indeed, at *WCS* and *Station 2*, the number of collected *Ae*. *albopictus* decreased as the distance from the forest edge toward the interior increased. Conversely, *Ae*. *gr*. *africanus* mosquitoes increased as the distance from the forest edge toward the interior increased (Fig 3B). Similarly, the relative abundance of both species varied according to the site and the degree of the wilderness. Indeed, the relative abundance of *Ae*. *albopictus* decreased with increasing wilderness (*p* = 0.04), whereas the relative abundance of *Ae*. *gr*. *africanus* increased with the wilderness (*p* < 0.001) (Fig 3C).

When we analyzed adult stages that emerged from the collected eggs, we observed a significant difference (Pearson’s Chi-square = 330.6, df = 2, *p* < 0.001) in the percentage of *Ae*. *albopictus* specimens for *WCS* (82.9%), *Station 1* (58.8%), and *Station 2* (9.9%) (Table 3). Moreover, the percentage of *Ae*. *albopictus* positive ovitraps (Table 4) differed significantly at the three sites (Pearson’s Chi-square = 46.1, df = 2, *p* < 0.001). These findings indicate that at *WCS* and *Station 1* ovitraps were colonized in similar proportions (Pearson’s Chi-square = 2.9, df = 1, *p* = 0.08), suggesting that *Ae*. *albopictus* presence was more associated with sites showing anthropogenic features.

**Table 4:** Ovitraps colonized by *Ae*. *albopictus*.

Site	Habitat	N. of traps deployed	N. of positive traps	%(C-interval)
WCS	Gallery	35	24	68.6 (50.7–83.1)
Station 1	Patch	29	26	89.6 (72.6–97.8)
Station 2	Forest	31	2	6.5 (0.7–21.4)

**C-interval**: Confidence interval of positive trap percentage; %: Percentage of positive traps

**Fig 4:**
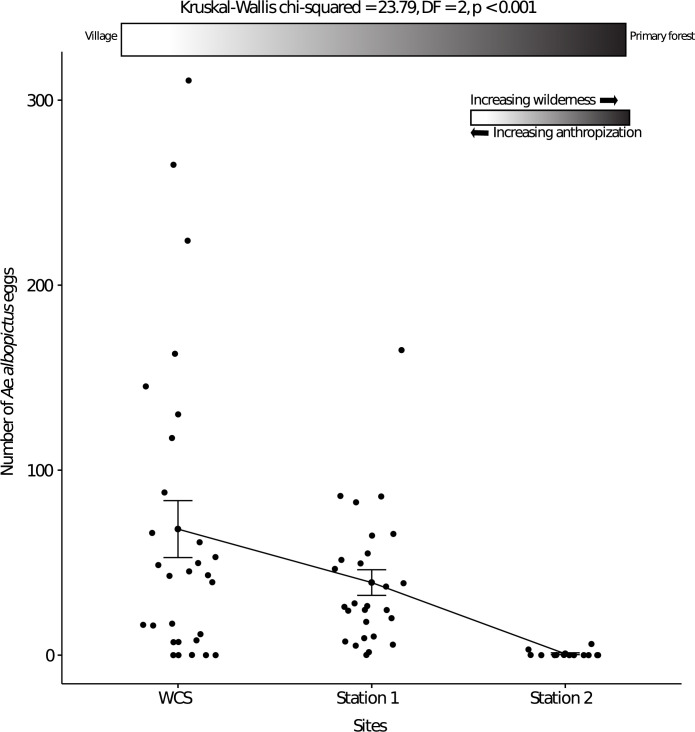
*Ae. albopictus* eggs laid in ovitraps along a decreasing gradient of anthropization. The gradient is from *WCS* (many human habitations) to *Station 2* (no human habitation).

We then used the percentage of *Ae*. *albopictus* adult specimens to calculate the estimated total WNAE collected at each site (defined as the frequency of *Ae*. *albopictus* emerged adults multiplied by the total number of eggs collected) (Table 3). Comparison of the WNAE values has shown that *Ae*. *albopictus* was the predominant species at sites with substantial anthropization (*WCS* and *Station 1*: characterized by the presence of human habitations with domestic or peri-domestic water containers). Conversely, it was weakly represented at the uninhabited site (*Station 2*). When we compared the mean number of *Ae*. *albopictus* eggs laid in each ovitrap among sites, we observed that this number significantly decreased (Kruskal-Wallis chi-squared = 23.79, df = 2, *p* < 0.001) from *WCS* (highly anthropized forest edge) to *Station 2* (lowly anthropized forest edge) (Fig 4).

### Model of penetration into the forest ecosystem

**Fig 5:**
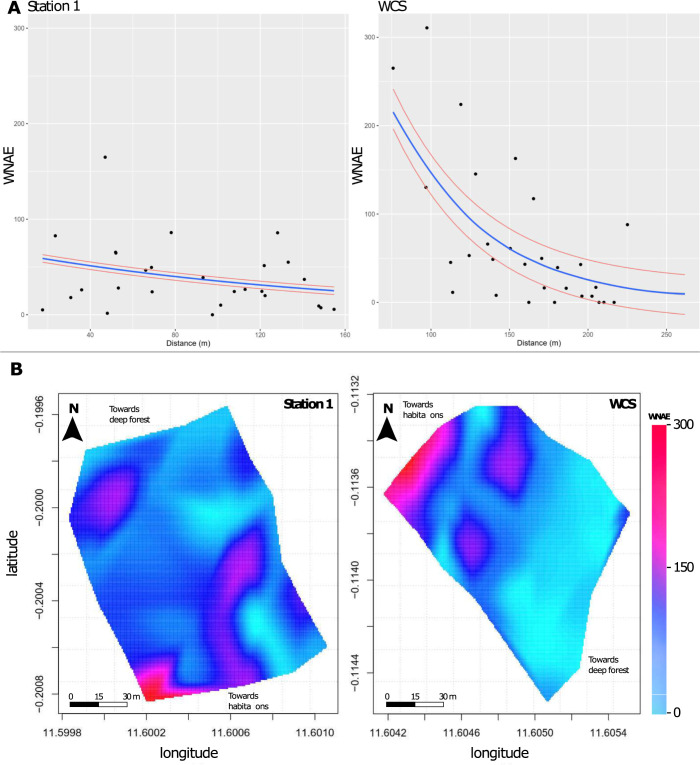
Models of *Ae*. *albopictus* penetration in the forest ecosystem from anthropized-wild environment interfaces’ margins. **A**: Scatterplot of the number of *Ae*. *albopictus* eggs in function of the distance from the forest fringe. Dots indicate the observed values and curves show the fitted negative binomial regression lines (± 95% confidence limits). The number of eggs in ovitraps progressively decreases with distance from the forest margin. **B**: Two-dimensional heatmap of *Ae*. *albopictus* egg density at *Station 1* and *WCS* showing density peaks at the forest margins close to human presence.

We evaluated *Ae*. *albopictus* colonization potential of the forest ecosystem by analyzing its oviposition activity at increasing distances from the forest fringe. The Shapiro-Wilk normality test (S2 Fig) showed that the WNAE values did not follow a normal distribution (W = 0.75, *p* < 0.001). We tested four different generalized linear (mixed) models with the residuals following the Poisson or the negative binomial distribution, and the “Habitat” variable included as a random effect, or otherwise without random effects. After model testing, we discarded the Poisson models because they showed data over-dispersion, and we focused on the negative binomial ones. We selected the best model using Likelihood Ratio tests (LR). The LR test indicated that random effects (*p* = 0.0002) and the "Distance" to the forest edge (z-value = -3.7, df = 52, *p* < 0.001) must be included in the minimal adequate model. The model parameters indicated that the number of *Ae*. *albopictus* eggs detected in the forest decreased progressively with the distance to the forest edge at both *Station 1* and *WCS* (Fig 5A). At *Station 1*, the mean number of eggs per trap decreased significantly from >50 at the forest edge to <10 beyond 100m from the forest edge (Fig 5A), with density peaks spatially concentrated along the forest edge (Fig 5B). Similarly, at *WCS*, the number of *Ae*. *albopictus* eggs decreased from >200 at the forest edge to <25 after 225m from the forest edge (Fig 5A), with density peaks spatially concentrated along the forest edge (Fig 5B). At *Station 2*, no clear pattern could be deduced due to the low number (n = 9) of specimens recovered (3 at 50m and 6 at 75m from the forest margin). These data showed that inhabited areas of forest fringes are the main *Ae*. *albopictus* entry points into wild forest ecosystems.

## Discussion

### Occurrence of sylvatic populations of *Aedes albopictus*

To the best of our knowledge, we report here for the first time a situation in continental Africa where *Ae*. *albopictus* occurs in a wild environment without established human presence. Indeed, our data highlight the multiple occurrences of this species at diverse locations throughout the LNP, up to 15km from the village, and its persistence over several years at sites distant from human habitations. These findings indicate that these *Ae*. *albopictus* populations might persist as sylvatic populations in areas where the human footprint anthropogenic influence varies from moderate (mainly limited to ecotourism and associated activities) to absent.

In the northern part of the LNP, we recorded *Ae*. *albopictus* occurrence at many sites located mostly in gallery forests across the savanna that interconnects the village to the primary forest at multiple locations southwards. These gallery forests, rarely exceeding 200m in width, might be highly suitable for this species, as previously described in similar environments in its native range in Asia [7] and in South-West islands of the Indian Ocean, such as La Réunion [38]. Potential natural larval breeding sites (rock holes and tree holes) and wild animals (as a blood source for adults) are abundant in these galleries. Thus, it seems likely that *Ae*. *albopictus* has gradually and actively progressed along these forest corridors from the village towards the primary forest. Moreover, its occurrence in fragments of isolated forest patches suggests that this mosquito actively crosses the savanna to colonize new areas, and/or disperse passively. Human activities in the park (e.g., ecotourism, conservation, and research) requiring cars transport from/to the village are a very effective means for passive displacement of *Ae*. *albopictus* adults even over long distances [39].

At La Lopé village (i.e., the anthropic compartment), the essential resources needed for *Ae*. *albopictus* life cycle are mainly provided by humans (i.e., blood sources and artificial water containers for larval development). However, as human presence decreases, the need to exploit alternative resources (wild animals and natural water containers) increases until it becomes the only option at sites without anthropogenic resources. The latter situation was observed at many sites throughout the northern part of the LNP suggesting that, *Ae*. *albopictus* exploited its opportunistic ecological preferences [18] to recover an ancestral sylvatic ecology [7]. Little is known about *Ae*. *albopictus* forest ecology in Africa, but we observed that natural breeding sites suitable for this species (mainly water-filled tree and rock holes) are abundant also in the forested environment of the LNP, in dense (gallery forests along rivers) and patchy (forest-savanna mosaic) areas. Nevertheless, the successful colonization of such natural microhabitats suggests that, *Ae*. *albopictus* has overcome several major biotic and abiotic constraints, demonstrating its adaptability. Biotic constraints include larval competition for space and food with the numerous resident mosquito species and other competing aquatic microorganisms. In Africa, tree holes host diversified communities of larval mosquitoes [40–42] that are shaped by interspecific competition, leading to species segregation in space and time to minimize niche overlap [43]. The colonization of tree holes by *Ae*. *albopictus* in the LNP probably implies complex interactions with native species, with possible species segregation. For example, in the LNP, we observed that, *Ae*. *albopictus* relative abundance increased progressively with the degree of anthropization, whereas that of *Ae*. *gr*. *africanus* decreased. Previous field and experimental observations in areas invaded by *Ae*. *albopictus* outside Africa clearly highlighted its ability to avoid interspecific competition and to coexist (or to displace) with resident North American mosquito species that lay eggs in tree holes, such as *Ochlerotatus triseriatus* [6] and *Ochlerotatus sierrensis* [44]. Alternatively, *Ae*. *albopictus* might exploit water-filled rock-holes for its larval development [18]. These microhabitats are extremely abundant in riverbeds or along the banks of streams in gallery forests in the northern part of the LNP. They also host a diversity of mosquito species communities [45], implying competitive interspecific interactions. The predation at natural larval development sites is an important issue that *Ae*. *albopictus* has to deal with, in regard to predators such as *Toxorhynchites* species (e.g., *T*. *brevipalpis*, *T*. *viridibasis*) that use tree holes for their breeding [46]. Other potential resident predators (e.g., arthropods and nematodes) and locally circulating pathogens (entomopathogenic viruses, fungi, and parasites) are additional biotic constraints that *Ae*. *albopictus* might overcome to successfully colonize natural breeding sites in forests that are not free spaces. Abiotic factors that might slow down *Ae*. *albopictus* spread in the sylvatic niche are linked to the environment and to rainfall that modulates the filling and also the flooding of natural larval development sites. In the northern part of the LNP, the rainfall pattern includes two drought periods (4 and 2 months) leading to temperature increase, relative humidity decrease and the progressive drying of many natural water containers. During these periods, several mosquito species tend to concentrate in a few number of large natural water collections where interspecific competition is probably exacerbated, or survive as diapausing eggs in dried containers waiting for the next rains. *Ae*. *albopictus* possesses this trait because it is an ancestral character of *Stegomyia* species. However, resident sylvatic species, which are its competitors, probably tolerate better egg desiccation [47]. It has already been suggested that differences in thermal and desiccation tolerance of eggs are key environmental drivers of the spatial and temporal coexistence between invasive *Ae*. *albopictus* and resident *Ae*. *aegypti* in Florida [48]. However, an experimental study demonstrated that desiccation resistance in non-diapausing eggs of *Ae*. *albopictus* is quickly selected [49]. Moreover, during extreme rain spells, most rock holes in riverbeds are rapidly flooded, causing important population crashes in mosquitoes that use this microhabitat. Overall, additional field and experimental studies on the ecology of pre-imaginal *Ae*. *albopictus* in forested environments are necessary to improve the knowledge in larval ecology, to define its ecological limits, and to assess its population dynamics.

### Penetration pattern of *Ae. albopictus* inside forest ecosystems

To better understand the potential for ecological niche expansion into the primary forest of *Ae*. *albopictus* sylvatic populations in the LNP, it is crucial to determine its capacity to survive and reproduce beyond the forest margins that have been proposed as its preferred habitat [7]. As our knowledge on the nature and abundance of natural larval microhabitats used by *Ae*. *albopictus* in African forests was limited, we used ovitraps to investigate its penetration and occupancy pattern in the forest ecosystem. We observed that the emergence success from egg to adult stage varied significantly in function of the collection site. Potential uncontrolled artifacts of egg conservation or larval growth in plastic trays might partly explain these differences. For example, wilting and collapse of eggs in some traps due to harsh desiccation during storage can hinder larval hatching and survival. However, the ovitrap survey carried out at the three sites showed a significant positive correlation between anthropization levels at the forest margins and *Ae*. *albopictus* relative abundance in ovitraps. By assuming that the availability of natural containers as breeding habitats was similar among sites, we hypothesized that the ovitrap attractiveness was homogenous among sites. Therefore, our result suggests that the *Ae*. *albopictus* population size (here approximated by egg density) remains strongly human dependent even in a sylvatic ecological context. Although it is not strictly required for its occurrence, the human presence contributes to maintaining *Ae*. *albopictus* density at the forest margins in the LNP, resulting in oviposition “hotspots” at these direct interfaces with humans (i.e., entry points). These findings also confirmed the close relation between *Ae*. *albopictus* and human settings from which females seems to move away only slightly. Anthropogenic water containers outside the forest could be more productive larval development sites compared with natural ones (their biotic and abiotic constraints for *Ae*. *albopictus* have been discussed above). Moreover, even if *Ae*. *albopictus* displays some trophic plasticity and feeds off a large vertebrate host spectrum, humans remain among the most frequent hosts [50]. Besides humans, domestic animals, when available, are more frequent blood sources than wildlife for *Ae*. *albopictus* collected deep in forests at anthropic-forest ecotones in Brazil [23].

In addition to the effect on egg density, we observed a continuous decrease of laid egg density from the forest margins (i.e., entry points) towards the deep interior of forest galleries. Indeed, most eggs were laid in the first 50m and then their number gradually decreased as the distance from the forest margin increased, becoming zero after 225m. In Brazil, where a similar study was carried out, the same oviposition pattern was observed. Specifically, the majority of *Ae*. *albopictus* eggs were recovered within the few hundred meters from the forest edge and up to 500m deeper in the forest [23]. This difference could be related to the higher mosquito population size at the forest margin in Brazil, due to a greater density of human habitations (up to 10 habitations/km^2^ in Brazil versus 2 habitations/km^2^ at most in the inhabited compartment of the LNP). In their study, Pereira *et al*. observed many traces of human passage over several hundred meters deeper and deeper into the forest [23]. Recurrent human movements in the forest might help *Ae*. *albopictus* to establish deeper in the forest because of its natural tendency to follow moving objects in their quest of blood [51]. The occupancy pattern we highlighted, with several spatially consecutive positive spots, suggests that, *Ae*. *albopictus* females gradually disperse from the margin into the forest ecosystem by successive short distances. Laboratory and field experiments indicate that, *Ae*. *albopictus* females practice skip oviposition that enhances its dispersal when looking for multiple oviposition sites during a single gonotrophic cycle [52]. The maximum distance we observed (225m) from the forest fringe conforms with the reported distance range actively traveled by *Ae*. *albopictus* during a single gonotrophic cycle in human settings [53]. Alternatively, dispersal deeper into the forest ecosystem could be related to other resource-seeking behaviors, including blood and sugar meals and resting sites [54,55]. Overall, our results indicate that *Ae*. *albopictus* has a limited aptitude to colonize forest ecosystems beyond their margins, and suggest that a permanent establishment in forests, in the absence of human occurrence, would remain associated with low population density, probably due to lower fitness in forest ecosystems, as discussed above. Regardless of population density, *Ae*. *albopictus* presence at the forest margins raises the question of its interactions with the wildlife present in the LNP, and of its potential role in the transmission of enzootic arboviruses.

### Sylvatic populations and potential risk of zoonotic transmission

*Ae*. *albopictus* propensity to evolve in natural ecosystems by exploiting natural larval breeding sites and by feeding on different animal species increases the risk of animal-to-animal and animal-to-human transmission of zoonotic pathogens [4]. Therefore, its colonization of natural ecosystems must be carefully monitored, especially in forested tropical areas where wildlife biodiversity and the risk of emerging infectious disease are high [56]. In areas of Central Africa invaded by *Ae*. *albopictus*, both *Ae*. *albopictus* and *Ae*. *aegypti* are commonly found in sympatry. However, studies investigating their distribution, especially in rural and forested settings and at the periphery of urban cities, reveal a trend towards the prevalence of *Ae*. *albopictus* over *Ae*. *aegypti* [57,58]. This suggests a progressive species replacement, especially due to the relatively recent introduction of *Ae*. *albopictus* to this area. This introduction has coincided with dramatic changes in the epidemiology of *Aedes*-borne viruses, with major outbreaks in cities and villages [12]. In some cases, new viral variants particularly well adapted to be transmitted by *Ae*. *albopictus* have been locally selected and exported to other areas colonized by *Ae*. *albopictus*, with an increasing risk of epidemics [59]. For example, in 2014, a CHIKV outbreak occurred in Montpellier (France) following its introduction from Cameroon [60]. Central Africa has become a source of *Ae*. *albopictus-*adapted arbovirus epidemic strains that can spread throughout and beyond Africa due to the increased flow of international travelers. The presence of *Ae*. *albopictus* in forest environments and its interactions with wild animals could further increase the risks of spillover and the emergence of *Aedes*-borne viruses from wildlife into the human compartment. Indeed, the occurrence of *Ae*. *albopictus* sylvatic populations in the LNP, sometimes in the absence of humans, implies that the species uses wildlife hosts as a source of blood [50], and might interact closely with the great diversity of enzootic viruses of the Congo Basin forests. Even if its spread were to be limited to forest fringes and gallery forests, interactions with wildlife would not be restricted. Indeed, such environments gathering savanna and diverse forest animals from diverse animal groups, are known for having an increased animal diversity/density [61], offering to *Ae*. *albopictus* many opportunities to feed on and to pick-up enzootic viruses. This continuous block of tropical forest (the largest after the Amazonian Basin) is a hot-spot for enzootic cycles, including arboviruses that involve sylvatic *Aedes* mosquitoes phylogenetically related to *Ae*. *albopictus* (i.e., all belonging to the *Stegomyia* subgenus) as vectors (Table 2). The LNP hosts a very abundant and highly diversified wildlife (>400 birds, 14 primates, 12 carnivores, 12 ungulate species, and >30 species of rodents and bats) [26] among which there are typical wildlife reservoirs of an extraordinary diversity of mosquito-borne enzootic viruses. For instance, nine species of monkeys present in the LNP (including 3 *Cercopithecus*, 1 *Cercocebus*, and 1 *Colobus*) are natural reservoirs of yellow fever, CHIKV, ZIKV and other arboviruses [62–66]. Repeated contacts between *Ae*. *albopictus* and some of the sylvatic vertebrate hosts (at least at sites where humans or domestic animals are absent) might promote interactions with some of the enzootic arboviruses suspected to be circulating in the LNP (Table 1). In this study, we primarily examined the level of penetration of *Ae*. *albopictus* in the sylvatic settings of the LPN, and we did not extensively explore its contact with wildlife and particularly with monkeys. To date, no data is available on potential contacts between *Ae*. *albopictus* and monkeys in the LNP. It would be important to investigate the interactions between *Ae*. *albopictus* and monkeys as well as their vertical distribution, to gain a more holistic understanding of the transmission dynamics. In addition, recent studies in tropical America forest ecosystems reported that *Ae*. *albopictus* might preferentially occupy the ground strata in the forest [67]. This suggests that in the LNP, *Ae*. *albopictus* might preferentially contact monkey species that exploit frequently or occasionally the ground strata. Moreover, our findings that *Ae*. *albopictus* continues to bite humans entering these forested areas suggests potential for a bridging role in the horizontal transfer of enzootic viruses between wildlife and humans. This risk already exists in La Lopé region, as indicated by the presence of bridge vector species, such as *Ae*. *africanus*; however, none of them thrives as *Ae*. *albopictus* does in the human compartment. This means that a virus picked up from wildlife and transmitted (i.e., selected) to humans by *Ae*. *albopictus* at the forest interface would have a higher probability to produce secondary cases once introduced in human settings because already adapted to *Ae*. *albopictus*. This potential risk should be defined more precisely by new surveys, including field-collected mosquitoes to identify the viruses carried by *Ae*. *albopictus* in sylvatic areas, and/or by vector competence studies, to assess its ability to transmit sylvatic virus strains that circulate among the wildlife in La Lopé region.

In conclusion, this study highlighted the penetration of the Asian tiger mosquito *Ae*. *albopictus* in the forest environment of Central Africa. We are witnessing a phase of invasion of the African forest ecosystem where sylvatic *Ae*. *albopictus* populations are mainly derived from human-associated populations. It remains to determine whether the possible selection of more zoophilic lineages that can survive and reproduce in the absence of humans will or has already occurred. From the One Health perspective, this introduction might increase the risk of transmission of zoonotic agents for which *Ae*. *albopictus* could play the role of a bridge vector, as previously hypothesized [7,23,68]. It is now crucial to determine *Ae*. *albopictus* capacity to exploit natural breeding sites in forest ecosystems (e.g., tree and rock holes) and to feed on wild vertebrates, and the effect of this invasion on the life cycles of sylvatic pathogens.

## Supporting information

S1 Fig Sampling plan diagram for ovitrap-based experiments. Sampling implemented along anthropization gradients at *WCS*, *Station 1*, and *Station 2* to assess *Ae*. *albopictus* penetration pattern inside forest ecosystems. Black full circles represent the position of individual ovitraps placed following five lines from the forest edge to the forest interior. The maximum distance from the forest edge varied according the site and the spatial conformation of the forest block (150m for *WCS*, 125m for *Station 1*, and 175m for *Station 2*).
(EPS)

S2 Fig Distribution of the weighted number of *Ae*. *albopictus* eggs (WNAE). The WNAE is highlighting a non-normal distribution.
(EPS)
